# Analysis of mortality predictors in a 1-year cohort of neuropalliative care patients

**DOI:** 10.1055/s-0046-1816036

**Published:** 2026-02-27

**Authors:** Diego Belandrino Swerts, Polyana Vulcano de Toledo Piza, Ana Luíza Araújo, Bernard Lobato Prado, Rafael Ferreira Docema, Caroline Miyake, Hye Sol Hwang, Mario Fernando Prieto Peres

**Affiliations:** 1 Hospital Israelita Albert Einstein, Faculdade Israelita de Ciências da Saúde Albert Einstein, São Paulo SP, Brazil.; 2 Hospital Israelita Albert Einstein, São Paulo SP, Brazil.

**Keywords:** Neurology, Palliative Care, Prognosis, Quality of Life, Palliative Performance Scale

## Abstract

**Background:**

Palliative care has been shown to yield benefits in terms of quality of life and therapeutic planning in oncological and non-oncological diseases, but its integration in neurology remains limited.

**Objective:**

To evaluate the predictive factors of mortality in neurological patients to improve prognosis.

**Methods:**

In this 1-year cohort study, we followed patients aged > 18 years hospitalized in a neurological Semi-Intensive Care Unit in Brazil. The patients were categorized into two groups based on palliative care indication: those with and those without indication. The palliative care criteria included a “no” response to the Surprise Question, a Palliative Performance Scale (PPS) score below 70%, or significant weight loss (> 5%) with associated body changes.

**Results:**

We included 166 patients, 87 with indication and 79 without it. The group with indication had a 29.8% mortality rate (26 deaths) and a 27.7-fold higher risk of death. The factors associated with increased mortality risk within 1 year included PPS score < 40%, answering “no” to the Surprise Question, and weight loss. Notably, 62% (n = 54) of the group with indication were admitted to the Intensive Care Unit (ICU), compared to 12% (n = 12) in the group without indication. Advanced directives were only documented in 31% (n = 27) of the group with indication and in 59% (n = 16) of the patients who died. Prognostic assessment in neurological diseases is challenging due to limited data.

**Conclusion:**

In the present study, we found that the PPS score, the Surprise Question, and weight loss were significant predictors of mortality within 1 year. These findings highlight the need for further research to provide better end-of-life markers and ensure greater autonomy in neurological disease management.

## INTRODUCTION

Palliative care (PC) is an approach to care for patients with serious illness aimed at improving the quality of life of them and their families. It is appropriate for patients at any stage of their illness, including at the time of diagnosis. 1 In neurology, there are several particularities in the definition of the criteria to indicate PC and in the care itself. Estimations of the duration of disease, the impact of the disease on a patient's functioning, and the possibility of relapse/recurrence of the disease may be difficult to predict. 2

Early identification of PC needs has demonstrated 3 benefits in quality of life, survival, and decision-making. As a result, prognostic tools are becoming increasingly common in PC. One key criterion in palliative prognostication involves asking the medical team the so-called *Surprise Question* : “Would you be surprised if the patient were to die in the next 12 months?”. A negative response has been shown to be the best predictor for PC indication and prognosis in oncological and non-oncological patients. 4 5 6 In a study involving oncological patients, a negative answer to surprise question was associated with a 7-fold increased risk of death within 1 year compared to the group who received an affirmative answer (hazard ratio [HR]: 7.787).

A study conducted in Argentina 7 aimed to identify predictive models of mortality in oncological patients, highlighting nutritional decline, decreased functionality (Palliative Performance Scale 8 [PPS] score < 50%), functional dependence, low therapeutic response, and persistent symptoms as key predictive factors; the use of the PPS showed a relative risk (RR) of 1.69 (95%CI: 1.35–2.13) for mortality.

In neurology, there is no widely-accepted definition in the literature regarding which patients should be indicated for PC. However, the discussion around PC in neurology has expanded following recommendations from the American Academy of Neurology. 9 Due to the relatively-recent nature of this debate, there are still few studies evaluating prognosis and survival in neurological palliative patients.

Given this gap, the primary objective of the current study is to evaluate the factors that increase the risk of 1-year mortality in neurological patients with an indication for PC. This assessment aims to improve prognostic evaluation and survival prediction in neurological palliative patients.

## METHODS

### Study design and participants

In the present cohort study, we included patients aged > 18 years who were hospitalized in the Neurological Semi-Intensive Care Unit (NSICU) at Hospital Israelita Albert Einstein, in the city of São Paulo, Brazil, between February and August 2022.

Data collection was conducted using convenience sampling, based on the availability of the researchers. However, on the days selected for data collection, efforts were made to evaluate all patients hospitalized in the NSICU. The patients were followed for 1 year from the time of data collection.

### Standard protocol approvals

The study was approved by the National Ethics in Research Committee of the Brazilian National Health Council through Plataforma Brasil (under CAAE 51792221.4.0000.0071), and by the Ethics Committee of Hospital Israelita Albert Einstein (under SGPP 4847-21). Informed consent was obtained from all participants or, in cases of incapacity, from their legal guardians (first-degree relatives or spouses).

### Eligibility criteria

The inclusion criteria were patients hospitalized due to a neurological condition or those with a neurological diagnosis admitted for complications related to their condition. Patients under 18 years of age, non-neurological patients, those admitted outside the researchers' available hours, and those who refused to participate were excluded.

Prior to the data collection, the researchers obtained the informed consent during hospitalization. The patients or their family members were approached at an appropriate and calm moment at the bedside in the NSICU to ensure a clear understanding of the study. Sufficient time was provided for them to accept or decline participation. Upon consent, the informed consent form was provided for signature. In the cases of incapacitated patients, informed consent was obtained from the patients' legal guardians.

The patients were followed up for 1 year, during which their medical records were reviewed, and calls were made using the contact information provided. The follow-up aimed to assess patient outcomes, including deaths and hospitalizations at Hospital Israelita Albert Einstein during the study period. Patients who had no recorded emergency visits or hospitalizations and did not respond to the follow-up calls were excluded; however, loss to follow-up was analyzed to minimize bias.

### Characteristics of the subjects

The patients filled out a questionnaire assessing demographic factors (such as age, sex, ethnicity, religion, and body mass index [BMI]), clinical and neurological diagnoses, duration of neurological illness, reason for hospitalization under PC, presence of advance directives, perception of health, and comorbidities (hypertension and diabetes). Functionality was evaluated using the PPS on the admission to the NSICU ( Table 1 ) and the Eastern Cooperative Oncology Group (ECOG) Performance Status (PS) scale. Data on the number of hospitalization days throughout the 1-year follow-up and patient survival status at 1 year were also collected. Furthermore, the following data were collected: location of death, PC team follow-up and, if so, the date of initiation, knowledge about PC, length of stay in the Intensive Care Unit (ICU), documentation of advanced directives in the medical records, use of the Surprise Question, and weight loss > 5%. The patients were categorized into two groups: those with and those without an indication for PC. The criteria for PC indication included a negative response by the researcher to the Surprise Question (‘Would you be surprised if the patient died within 12 months?’), a PPS score below 70%, or weight loss greater than 5% accompanied by noticeable physical changes reported by the patient or family members ( Table 2 ).

**Table 1:** Palliative Performance Scale 8

%	Ambulation	Activity and evidence of disease	Self-care	Intake	Level of consciousness
**100**	Full	Normal, no evidence of disease	Complete	Normal	Complete
**90**	Full	Normal, some evidence of disease	Complete	Normal	Complete
**80**	Full	With effort, some evidence of disease	Complete	Normal	Complete
**70**	Reduced	Unable to work, some evidence of disease	Complete	Normal or reduced	Complete
**60**	Reduced	Unable to perform hobbies, significant disease	Occasional assistance	Normal or reduced	Fully alert or with periods of confusion
**50**	Mainly sit/lie	Incapable of any work, extensive disease	Considerable assistance	Normal or reduced	Fully alert or with periods of confusion
**40**	Mainly in bed	Incapable of any work, extensive disease	Almost complete assistance	Normal or reduced	Fully alert or with periods of confusion
**30**	Bed bound	Incapable of any work, extensive disease	Complete dependence	Reduced	Fully alert or with periods of confusion
**20**	Bed Bound	Incapable of any work, extensive disease	Complete dependence	Limited intake to spoonfuls	Fully alert or with periods of confusion
**10**	Bed Bound	Incapable of any work, extensive disease	Complete dependence	Mouth care	Confused or comatose
**0**	Death	–	–	–	

**Table 2:** Major ANCP criteria 14 adapted

Primary criteria
Question: Would you be surprised if the patient were to die within 12 months?
Weight loss greater than 5% accompanied by noticeable physical changes
PPS score < 70%

Note: Tavares de Carvalho R, Afonseca Parsons H, (organizadores). Manual de Cuidados Paliativos ANCP Ampliado e atualizado. Acad Nac Cuid Paliativos. 2012;1–592.

### Outcomes

The primary objective of the current study is to evaluate predictors of mortality, including nutritional decline, PPS score, the Surprise Question, and family perception among neurological patients. Family perception was assessed by asking about the patient's general health: ‘How do you rate your/the patient's health?”; the possible answers were *good* , *really good* , *regular* , *bad* , and *really bad* .

The secondary objectives were to assess whether the indication group presented higher mortality rates compared to those without an indication throughout 1 year, and to determine whether the indication group experienced more hospitalizations and spent more days in the ICU. The frequency of advanced-living directives documented on the medical records was also evaluated.

### Statistical analysis

For sample size determination, we considered the survival results reported by Moss et al., 6 specifically comparing survival between patients in the ‘No’ and ‘Yes’ groups regarding the answer to the question, a recognized predictor of an indication to PC ( Table 3 ). A sample calculation was performed using the logrank test and HR data for the Surprise Question. Assuming a power of 80%, a significance level of 5%, and a 5% rate of loss to follow-up in each group, and using the proportions found in the Moss et al study (16% in the ‘No’ group and 84% in the ‘Yes’ group), an HR of 7.787 was detected, with 32 patients in the indication group indication, leading to a total sample size of 198 patients. These calculations were performed using the Power Analysis and Sample Size (PASS; NCSS, LLC) software. 10 Based on this sample, if the rate observed by Moss et al. 6 was maintained, we expected to identify 31 palliative cases, enabling the adjustment of multiple logistic models with up to 3 explanatory variables. 11 12

**Table 3:** Comparison of demographic and epidemiological characteristics of the groups with and without an indication for palliative care

Characteristic		Without indication	With indication	*p* -value
		(79)	(87)	
**Mean age (years)**		59.10 ± 18.44	74.92 ± 18.45	< 0.05 a
**Male sex (%)**		48.7	43.6	0.36 b
**Diabetes (%)**		15.1	28.7%	< 0.05 b
**Arterial hypertension (%)**		39.2	51.1	0,12 b
** Mean Body Mass Index (kg/m ^2^ ) **		26.25 ± 5.97	24.99 ± 5.34	0.012 a
**Ethnicity (%)**	White	93.6	94.18	0.99 b
	Brown	5	4.6	
	East and Central Asia	1.2	1.1	
**Religion (%)**	Catholic	59.7	63.4	0.84 b
	Evangelical Christian	6.4	2.4	
	Spiritualistic	3.8	6	
	Jewish	10.3	9.7	
	Atheist	9	8.5	
	Others	10.3	9.7	
**Neurological diagnosis (%)**	Neurovascular diseases	41.7	31.03	0.02 b
	Neurodegenerative diseases	5	0	
	Syndrome of dementia	7.5	25.28	
	Parkinson's disease	0	4.5	
	Epilepsy	8.8	4.5	
	Anoxic brain injury after cardiac arrest	0	1.1	
	Primary neural cancer	8.8	6.8	
	Brain metastasis	1.2	2.2	
	Neuromuscular diseases	2.4	8	
	Neuroinfectious diseases	4.8	2.2	
	Primary headaches	6	0	
	Trauma	12.6	13.7	
**Performance status (%)**	Really bad	1.3	8.3	< 0.05 b
	Bad	9.2	20.23	
	Regular	31.5	38.09	
	Good	36.8	28.57	
	Really good	21.05	4.7	

Notes: ^a^ Chi-squared test; ^b^ Kruskal wallis test.

The data collected was compiled in an Excel (Microsoft Corp.) spreadsheet and subsequently analyzed using the R Commander platform (free and open-source). The sample was characterized using mean, standard deviation, minimum and maximum, median, and quartile values for the quantitative variables, while absolute and relative frequencies were used for the qualitative variables. 13 The data was presented in tables and graphs. The baseline conditions of the patients were categorized by neurological diagnosis, presence or absence of PC indication, and further analyzed by categorical variables such as gender and quantitative variables such as age and duration of illness.

The demographic and clinical characteristics of the two groups were compared through the Chi-squared test or the Fisher's exact test for the categorical variables, and Student's *t* -test or the Kruskal-Wallis test for the continuous variables, depending on data distribution. Normality was assessed using the Shapiro-Wilk test, box plots, histograms, and quantile-quantile plots. 13 Values of *p* < 0.05 were considered statistically significant.

We conducted a comparative analysis of patient survival based on several key characteristics, including: PC indication (yes/no), recent significant weight loss (> 5% of the previous weight; yes/no), patient health status (really bad, bad, regular, good, or really good), answer to the Surprise Question (yes/no), PPS score, ECOG PS score, length of hospital stay, sex, age, religion, ethnicity, and ICU admission within the previous 30 days.

In the multivariate model for 1-year survival, we included all variables that demonstrated significance at the 10% level in the univariate analysis. Two distinct models were developed based on predefined cut-off points: model 1 compared patients with PPS scores > 40 and ≤ 40, while model 2 compared those with PPS scores > 70% and ≤ 70%.

The survival function was estimated using the Kaplan-Meier method and the relationship regarding the variables of interest and survival time was assessed using Cox proportional hazards regression models. All analyses were conducted using the IBM SPSS Statistics for Windows (IBM Corp.) software, version 24.0, with a significance level of 5%. In cases of missing data, the researchers attempted to retrieve information from electronic medical records. If data remained unavailable, it was excluded from the analysis.

## RESULTS

Throughout the study, 247 neurological patients were admitted to the NSICU, with 198 patients included, corresponding to 80% of the sample. There were 5 refusals, and 44 patients were not included due to incompatibility between the admission date and the researchers' data collection schedule. Additionally, 10 patients from the initial analysis were assessed via telephone or medical records but had missing data regarding weight, race, symptoms on the Edmonton scale, and health perception by the medical team. These patients were excluded from the prospective cohort. Of the remaining 188 patients, 9 lacked a definitive diagnosis and were excluded from the sample. Ultimately, 179 patients remained, but 13 were lost to follow-up, as they did not respond to phone calls or attend a consultation 1 year after data collection. These missing patients included 6 from the indication group and 7 from the no indication group, with 4 scoring < 50% on the PPS.

The demographic characteristics of the study population are summarized in Table 3 . The key differences between the indication and no indication groups include: a slightly-higher proportion of male patients in the no indication group (48.7% versus 43.6%); a significantly-higher mean age in the indication group (74.92 ± 18.45 years versus 59.10 ± 18.44 years); and a lower BMI in the indication group. In terms of comorbidities, 28.7% of the indication group had diabetes, and 51% had arterial hypertension. The sample was predominantly White (94%), and religiosity was similar between the 2 groups, with Catholicism being the most common affiliation.

Of the 166 patients included in the final analysis, 87 (52%) met the criteria for the indication group. Among them, 75 had a PPS score < 70%, 50 (30%) received a negative prognosis from the researchers, and 36 (21%) had a weight loss greater than 5% associated with noticeable changes in body appearance, as reported by family members. Furthermore, 44 patients had a PPS score ≤ 40%. Regarding neurological diagnoses, dementia and trauma were more prevalent in the indication group, although they were also among the most common conditions overall. The distribution of neurological diagnoses was as follows: 36% (n = 59) had neurovascular disease, 16.8% (n = 28) had dementia, 6.6% (n = 11) had epilepsy, 7.8% (n = 13) had primary brain tumors, 13.2% (n = 22) had traumatic brain injury, 3.6% (n = 6) had neuroinfectious diseases, and 20% had other neurological pathologies, reflecting a diverse sample.

**Figure 1:**
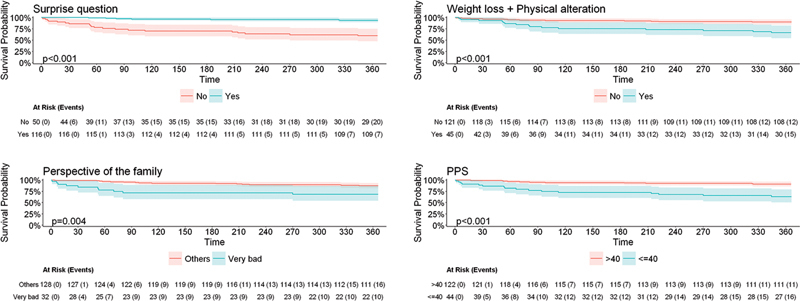
Kaplan-Meier survival curve regarding different scales and tools in 1 year.

Within 1 year, 27 patients died, 26 of whom belonged to the indication group (29.8% of mortality in this group), with *p* < 0.01 compared to the group without indication. We found evidence of associations involving survival and all variables of interest, except for length of hospital stay ( *p* = 0.267; Table 4 ). The patients in the indication group had a higher risk of death (HR = 27.783; *p* = 0.001), as did those with recent unexplained weight loss (HR = 3.794; *p* = 0.001), the subjects with a negative answer to the Surprise Question (HR = 8.46; *p* < 0.001), the participants whose family members rated their condition as *very bad* or *bad* (HR = 3.027; *p* = 0.006), and the patients with ECOG PS III or IV (HR = 5.11; *p* < 0.001). The PPS score was evaluated using the cut-off points of ≤ 40% and ≤ 70%, also showing an increased risk of patient death (HR = 4.885; *p* < 0.001; and HR = 4.863; *p* = 0.004 respectively) ( Figure 1 ).

**Table 4:** Tools and scales associated with 1-year survival

Variables (n)		Death (n)	HR (95%CI)	*p* -value
Indication for palliative care	Yes (87)	26	27.783 (3.769–204.811)	**0.001**
	No (79)	1		
Have you/the patient recently experienced unexplained weight loss of more than 5% of your/the patient's previous weight?	Yes (36)	15	3.794 (1.775–8.110)	**< 0.001**
	No (130)	12		
Surprise Question	No (50)	20	8.460 (3.572–20.037)	**< 0.001**
	Yes (116)	7		
Family's perception of the patient's health	Bad/Very bad (32)	10	3.027 (1.373–6.674)	**0.006**
	Others (128)	16		
PPS score	≤ 40% (44)	11	4.885 (2.265–10.537)	**< 0.001**
	> 40% (122)	16		
	≤ 70% (75)	4	4.863 (1.681–14.066)	**0.004**
	> 70% (91)	23		
ECOG PS score	III–IV (73)	21	5.110 (2.062–12.665)	**< 0.001**
	0–II (93)	6		
Age			1.025 (1.002–1.049)	**0.032**
Days hospitalized			1.003 (0.998–1.007)	0.267

Abbreviations: ECOG PS, Eastern Cooperative Oncology Group Performance Status scale; HR, hazard ration; PPS, Palliative Performance Scale.

Table 5 presents the results of the adjusted multiple models, which considered all significant variables at the 10% level. Two models were developed based on different PPS cut-off points: model 1 (> 40% versus ≤ 40%) and model 2 (> 70% versus ≤ 70%). Both models showed that PC indication and the Surprise Question were significant predictors of mortality. Specifically, patients in the indication group had a substantially-higher risk of death, with an HR of 17.6 ( *p* = 0.012) in model 1, and an HR of 16.1 ( *p* = 0.017) in model 2. Additionally, patients whose family members perceived their health as *bad* or *really bad* had a significantly higher risk of mortality, with HRs of 3.36 ( *p* = 0.027) and 3.60 ( *p* = 0.022) in models 1 and 2 respectively.

**Table 5:** Multiple models for 1-year survival

Variables		PPS score < 40%		PPS score < 70%	
		HR (95%CI)	p-value	HR (95%CI)	*p* -value
Indication for palliative care	Yes	17.610 (1.872–165.669)	**0.012**	16.139 (1.640–158.855)	**0.017**
	No	Reference	–	Reference	–
Have you/the patient recently experienced unexplained weight loss of more than 5% of your/the patient's previous weight?	Yes	1.653 (0.726–3.766)	0.232	1.683 (0.741–3.823)	0.214
	No	1	–	1	–
Surprise question	No	3.356 (1.149–9.803)	**0.027**	3.690 (1.207–11.277)	**0.022**
	Yes	1	–	1	–
Family's perception of the patient's health	Bad | Very Bad	1.678 (0.708–3.976)	0.240	1.755 (0.747–4.124)	0.197
	Others	1	–	1	–
PPS score	≤ CP	1.398 (0.491–3.982)	0.531	1.184 (0.309–4.537)	0.805
	> CP	1	–	1	–
ECOG PS score	III–IV	0.361 (0.096–1.356)	0.131	0.407 (0.111–1.493)	0.175
	0–II	1	–	1	–
Age		0.996 (0.970; 1.023)	0.765	0.995 (0.968–1.023)	0.732

Abbreviations: ECOG PS, Eastern Cooperative Oncology Group Performance Status scale; HR, hazard ration; PPS, Palliative Performance Scale.

Notes: CP: cutoff point; Model 1–40; model 2–70; *p* -value for the Cox regression model.

Among the 87 patients in the indication group, 31% (n = 27) had advanced directives recorded in their medical records. Of the 27 patients who died, 59% (n = 16) had advanced directives. However, only 37% (n = 13) of the deceased patients received an evaluation by the PC team.

Regarding ICU admissions, within 1 year, 62% (n = 54) of the patients in the indication group were admitted to the ICU, compared to 12% (n = 12) of the patients in the no indication group ( *p* < 0.01). The mean length of ICU stay was also significantly longer in the indication group compared to the one without indication (8.96 days versus. 2.27 days respectively; *p* < 0.01). Furthermore, the patients in the indication group spent an average of 58 days hospitalized throughout the year, compared to 14 days in the no indication group ( *p* < 0.01). Of the 27 patients who died, 13 died in the ICU, 14 died in the hospital ward, and 1 patient died outside the hospital. Among those in the indication group, 11 (40%) died in the ICU.

## DISCUSSION

In 2011, Academia Nacional de Cuidados Paliativos 14 (ANCP, the Brazilian National Academy for Palliative Care) established criteria to identify patients who would benefit from PC upon admission, and one of the key indicators was a negative response to the Surprise Question. 4 5 Additionally, factors such as recurrent hospitalizations, difficulty managing symptoms, declining functionality, need for life-sustaining interventions, and unexplained weight loss were also considered.

In neurology, given the different diagnoses and disease progressions, the timing of PC team involvement is not well-defined. The division of patients into four groups has been proposed: patients with rapid decline, those with episodic decline, those with prolonged decline, and patients with acute crises and uncertain improvement. Early identification of PC needs has demonstrated benefits in quality of life, survival, and decision-making.

There is great difficulty in defining prognosis and survival in medicine; in the field of neurology, there are few studies that evaluate scales and methods for prognostic assessment. In previous studies, 15 16 there was a correlation between the PPS and patient mortality at 1 year, both in oncological and non-oncological PC patients. In our cohort, we assessed how some of the well-known tools for prognostic prediction perform in the prediction of 1-year mortality of patients with neurological disease in an NSICU.

Prognostic assessment is essential for several reasons: to respect patient autonomy, to gather their preferences regarding treatment and goals of care, and to guide end-of-life treatment decisions. Furthermore, adequate prognostic assessment has been shown to be related to reduced psychological distress and improved quality of life. Clear communication about prognosis and goals of care is crucial to respect patient autonomy. Additionally, patients who are well-informed about their illness report lower levels of anxiety and higher health-related quality of life scores. 17 18

However, prognostic assessment in neurological diseases remains challenging due to the limited availability of reliable data. Despite the varied subgroups in the cohort of the current study, the aim was to include neurological patients with different diseases to evaluate simple tools validated in other specialties for the prognostication of those patients, expanding the debate on indications for PC in neurology. In the present study, the PPS and ECOG PS scores, the use of the Surprise Question, weight loss associated with body changes, and family perception of the patient's health were statistically significant predictors of mortality in neurological patients over a 1-year period. The PPS score may have been influenced by acute worsening of the condition. Therefore, the PPS score reflects an isolated value at the time of collection by researchers, which is a limitation of the study, since it does not necessarily reflect the PPS score in the out-of-hospital setting.

In a previous review 19 of patients with hemorrhagic stroke, for example, factors such as age, clinical examination on admission, hemorrhage volume, comorbidities, bleeding location, initial intracranial hemorrhage severity, and previous anticoagulation use were found to be unreliable predictors of prognosis. Similarly, in studies following cardiorespiratory arrest, 20 the prognosis was moderately predicted by pupillary response 72 hours after the event and the evaluation of evoked potentials.

Palliative care is particularly challenging for patients with brain tumors, as the presentation varies greatly across different subtypes, and patient behavior can differ depending on the primary site of metastasis. 21 Consequently, prognosis in neurology research lacks both quality and quantity. 22 The current study, which included a heterogeneous sample of neurological diseases, represents a step forward in developing a better prognosis for these patients using clinical criteria alone.

A study 23 conducted in São Paulo found that most patients admitted to the NSICU were indicated for PC. Patients in the indication group presented a higher prevalence of symptoms such as fatigue, depression, shortness of breath, and lack of appetite, and required more supportive interventions, including oxygen therapy, enteral or parenteral nutrition, ICU admissions, and longer hospital stays. In our cohort, patients in the indication group presented a relative risk of death 23 times greater than those without an indication, illustrating the reliability of the adopted criteria to discuss therapies and directives with patients.

Between 2000 and 2006, a study 24 in the United States highlighted that many elderly Americans required decision-making support at the end of life, often when they lacked the capacity to make decisions. Patients who had prepared advance directives received care that closely aligned with their preferences. In the current study, only 29% of the patients in the indication group had advance directives documented, underscoring the need to clearly define PC criteria for neurological patients and to improve prognostic indicators for this population. Of the patients who died, 59% had advance directives recorded on their medical records.

Thus, like in other specialties, the present study validates these tools, associating them with clinical data, family perception, and the treatments available for the patients, adding more tools for prognostication and contributing to the discussion on PC indication in these patients, as well as earlier follow-up with the PC team. In the current study, we did not consider patient treatment, as the objective was to evaluate predictor scales, regardless of treatment or neurological condition, to assess whether there was an increased risk of death in 1 year at a tertiary hospital.

As a limitation, the study did not show heterogeneity regarding race and socioeconomic profile, with difficulty in external validation in a population with a different socioeconomic profile. However, given the fact that the socioeconomic profile of the sample of the current study was above the Brazilian average, we believe that prognostic tools tend to carry an even higher relative risk. Thus, the present study broadens the discussion about the need for more research into prognosticating neurological patients, to provide better end-of-life markers, earlier follow-up with the neuropalliative care team, and ensure greater autonomy for individuals facing neurological diseases.
